# Treatment of exceptionally large prostate cancer patients with low‐energy intensity‐modulated photons

**DOI:** 10.1120/jacmp.v7i4.2263

**Published:** 2006-11-28

**Authors:** Mei Sun, Lijun Ma

**Affiliations:** ^1^ University of Maryland School of Medicine Department of Radiation Oncology Baltimore Maryland U.S.A.

**Keywords:** IMRT, prostate cancer, treatment planning

## Abstract

An inverse planning technique using 6‐MV intensity‐modulated photon beams was developed for treating large‐size patients with prostate cancer. Comparisons of treatment plans using 6‐MV and 18‐MV intensity‐modulated beams were carried out for a cohort of 10 patient cases. For these cases, we analyzed the dependence of plan quality on the beam energies. We found that 6‐MV beams resulted in plans equivalent to those for 18‐MV beams both for targets and for critical structures such as the rectum and bladder. The differences between the plans in the integral dose and the mean dose to the normal tissue surrounding the target were found to be small, in contrast to those for 3D conformal plans. Our findings showed that the low entrance dose of the high‐energy photon beams is mostly compensated by the high exit dose for even exceptionally large patients. In conclusion, 6‐MV intensity‐modulated beams are a feasible choice for treating large‐size patients with prostate cancer, provided that proper inverse planning techniques are adopted.

PACS number: 87.50.Gi, 87.53.Tf

## I. INTRODUCTION

Intensity‐modulated radiation therapy (IMRT) has been widely used for prostate cancer treatments.^(^ ^1^ ^–^ ^15^ ^)^ In practice, high‐energy photons such as 18 MV are often used, given the experience of 3D conformal radiation therapy with static beams. However, in IMRT treatments, the effects of the intensity modulation and the use of a relatively large number of beams has been found to reduce the dependence of the treatment planning on the selection of beam energies.^(16)^ As a result, 6‐MV photon beams have been found to be an effective energy choice for most IMRT cases.^(^ ^17^ ^,^ ^18^ ^)^

In addition, the total monitor units are typically two to three times higher in IMRT than in conventional radiation therapy. Therefore, the use of high‐energy photons also raised concerns about increased leakage and secondary neutron dose for the patients.^(^ ^19^ ^–^ ^21^ ^)^ However, it is unclear whether low‐energy intensity‐modulated photons can be used for large‐pelvis irradiations because of the low penetration power of the beam.

At our institution, we often encounter large‐size patients having prostate treatments. About 20% of our patients have vertical or lateral separations of more than 25 cm. In the present study, we aimed to develop an inverse planning technique and to investigate the feasibility of using 6‐MV intensity‐modulated photons for treating exceptionally large patients (vertical or lateral separations > 30 cm) with prostate cancer. The benefits of using 6‐MV instead of 18‐MV photons include elimination of secondary neutron production and reduction in room shielding burden. Additionally, 6 MV is widely available for single‐modality linear accelerators, potentially making IMRT more accessible to the population of larger‐size patients.

## II. MATERIALS AND METHODS

A cohort of 10 prostate cases was selected for our study. The mean anterior–posterior (AP) separation of these patients was 31 cm (range: 25–34 cm) and the mean lateral separation was 41 cm (range: 35–46 cm). Of these patients, 6 received full‐course IMRT treatment, and the other 4 received IMRT boost treatments following whole‐pelvis irradiations of 45 Gy. The average planning target volume (PTV) for these patients was 263 cc (range: 155–302 cc).

For inverse IMRT treatment planning, we used a 7‐coplanar‐beam arrangement at 0‐, 51‐, 102‐, 153‐, 207‐, 258‐, and 309‐degree gantry angles following published techniques.^(^ ^3^ ^,^ ^9^ ^,^ ^15^ ^)^ Those studies showed that 7 beams are advantageous over numbers such as 5 or 9 for effective and efficient prostate IMRT treatments.

**Figure 1:**
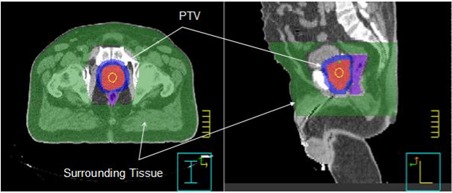
Illustration of the normal‐tissue region where the dose–volume constraints are imposed. PTV=planning target volume

The plan was generated on a commercial treatment planning system (Pinnacle^3^ v.7.4, Philips Medical, Milpitas, CA). For all plans, we defined the dose–volume constraints for a surrounding normal tissue region in addition to the dose–volume constraints for the target volume and normal structures such as rectum and bladder. Fig. 1 illustrates the definition of the normal‐tissue region.

As illustrated in the figure, the normal tissue typically extends from the boundary of the PTV plus 1‐cm margin to the skin surface. We imposed the dose–volume constraint on this volume such that no more than 25% of the surrounding normal‐tissue volume would receive 40% of the prescription dose. This additional dose–volume constraint is the key to producing a conformal dose distribution around the PTV and limiting dose “streaking” effects–e.g., high‐dose areas near the beam entrances. The streaking effects are typically more pronounced for the 6‐MV intensity‐modulated photons in these large‐size patients.

For all of the cases, we developed treatment plans using 6‐MV and 18‐MV intensity‐modulated beams with identical dose–volume constraints. The dose–volume histograms (DVHs) for the 6‐MV and 18‐MV plans were compared for the target volumes [gross tumor volume (GTV) and PTV] and for critical structures such as the rectum and the bladder. Because the PTV overlaps with the rectum and the bladder in all cases, the coverage of the PTV is constrained such that 100% of the PTV receives at least 95% of the prescription dose.

We also defined the conformal index to compare the treatment plans. The conformal index is defined as the ratio of the 95% isodose volume divided by the PTV volume that is enclosed by the 95% isodose line. From this definition, the closer the conformal index approaches 1, the more conformal is the treatment plan.

We also calculated the integral dose for the rectum, the bladder, and the surrounding normal tissue by integrating the dose over all voxels within each volume. The mean dose is defined as the integral dose divided by the volume of interest. From the definition, the unit of the integral dose is *J*, and the unit of the mean dose is Gy, assuming unit tissue density.

## III. RESULTS AND DISCUSSION

**Figure 2:**
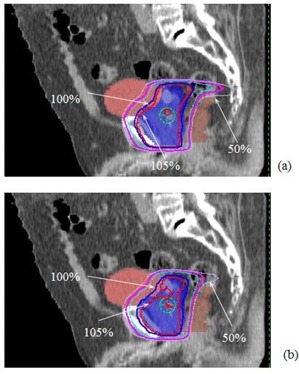
Illustration of the effects of the surrounding normal‐tissue constraints imposed on intensity‐modulated radiotherapy dose distributions: (a) the dose distribution without surrounding normal tissue constraints; (b) the dose distribution with the surrounding normal‐tissue constraints. Note the reduction of the dose streaking effect at the 50% isodose line

Fig. 2 illustrates the effects of the dose–volume constraints imposed on the surrounding normal tissue. The result shows the reduction of the peripheral dose streaking areas, as well as the dose hot spots inside the target.

In general, we found that a more uniform dose for the target volume can be achieved when the surrounding normal‐tissue constraints are imposed. This finding suggested degeneracy in the inversely planned solutions. Adding normal‐tissue constraints therefore helps to avoid the local minimum or suboptimal solutions for the problem.

**Figure 3:**
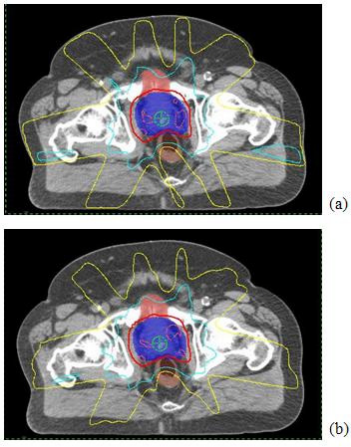
Comparison of the axial dose distributions between the 6‐MV and 18‐MV intensity‐modulated radiotherapy treatment plans for a large patient with prostate cancer: (a) the 6‐MV plan; (b) the 18‐MV plan

**Figure 4:**
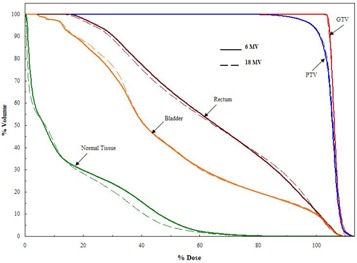
Comparisons of the dose–volume histograms for 6‐MV (solid line) and 18‐MV (dashed lines) intensity‐modulated radiotherapy plans for the case shown in Fig. 3

Fig. 3 shows the isodose distribution for a typical patient case, and Fig. 4 gives the dose– volume histogram (DVH) comparison of the 6‐MV and 18‐MV treatment plans. From the results in Figs. 3–4, 6‐MV and 18‐MV beams yielded nearly identical dose distributions for GTV, PTV, rectum, and bladder. The maximum dose inside the PTV is slightly better (~1%) for the 6‐MV treatment plan than for the 18‐MV plan. The conformal index is identical between the two treatment plans. The surrounding normal‐tissue dose is similar, with the DVH curve for the 6‐MV plan being slightly higher near the low‐dose region, but lower near the high‐dose region. As a result, the mean dose normalized by the prescription dose is 21% for the 6‐MV plan and 20% for the 18‐MV plan. Such a small difference indicates that the low entrance dose from the high‐energy beam is, in effect, compensated by the high exit dose.

Table 1 gives a summary of the results for all the studied cases, listing the dose delivered to part of the critical structure volume such as 1/3, 2/3 of bladder and rectum. The dose values in the table are all normalized to the prescription dose. For rectum and bladder alike, the difference between 6 MV and 18 MV falls within 1%−2% of the prescription dose. In general, the dose to the 1/3 of the rectum volume (i.e., the high‐dose area) is slightly higher for the 18‐MV plan, and the dose to the 2/3 of the rectum volume (i.e., the low‐dose area) is slightly lower for the 6‐MV plan. On average, the 6‐MV and 18‐MV plans are equivalent with regard to the dose to rectum and bladder, as shown in Table 1.

**Table 1:** Dosimetric parameters for all the cases, comparing 6‐MV (6x) with 18‐MV (18x) treatment plans for large patients with prostate cancer, where $V_{R}$ denotes the rectum volume; $V_{B}$ denotes the bladder volume; $V_{N}$ denotes the normal tissue volume, CI denotes the conformai index, 6x denotes 6‐MV photon beam, and 18x denotes 18‐MV photon beam

	Mean dose (normalized to prescription dose)						Dose to fractional structure volume (normalized to prescription dose)															
	Rectum		Bladder		Normal tissue		$D(1/3\;V_{R})$		$D(2/3\;V_{R})$		$D(1/3\;V_{B})$		$D(2/3\;V_{B})$		$D(1/3\;V_{N})$		$D(2/3\;V_{N})$		CI		MU	
Case	6×	18x	6×	18x	6×	18x	6×	18x	6×	18x	6×	18x	6×	18x	6×	18x	6×	18x	6×	18x	6×	18x
1	0.63	0.63	0.53	0.53	0.21	0.20	0.80	0.81	0.48	0.47	0.75	0.75	0.30	0.30	0.32	0.28	0.07	0.10	1.31	1.31	620	498
2	0.51	0.51	0.48	0.48	0.19	0.18	0.64	0.63	0.32	0.31	0.55	0.54	0.30	0.30	0.27	0.24	0.08	0.09	1.41	1.41	556	450
3	0.65	0.66	0.59	0.59	0.13	0.12	0.75	0.75	0.51	0.52	0.76	0.75	0.44	0.43	0.13	0.13	0.02	0.02	1.35	1.35	441	361
4	0.48	0.48	0.62	0.62	0.16	0.15	0.59	0.60	0.39	0.38	0.76	0.77	0.46	0.45	0.22	0.20	0.05	0.06	1.19	1.23	420	352
5	0.65	0.65	0.50	0.50	0.11	0.10	0.83	0.84	0.48	0.47	0.57	0.57	0.33	0.35	0.15	0.15	0.02	0.02	1.32	1.33	552	497
6	0.45	0.45	0.47	0.46	0.17	0.16	0.60	0.60	0.23	0.21	0.60	0.59	0.22	0.21	0.21	0.19	0.04	0.05	1.26	1.28	608	537
7	0.62	0.61	0.91	0.90	0.18	0.17	0.79	0.79	0.42	0.41	0.10	0.10	0.86	0.85	0.26	0.23	0.05	0.07	1.34	1.35	562	512
8	0.82	0.82	0.75	0.75	0.16	0.15	0.94	0.95	0.75	0.74	0.90	0.91	0.66	0.65	0.25	0.22	0.05	0.05	1.35	1.36	433	364
9	0.55	0.55	0.32	0.32	0.18	0.16	0.69	0.68	0.38	0.37	0.04	0.04	0.06	0.05	0.25	0.22	0.06	0.07	1.23	1.27	620	458
10	0.41	0.40	0.19	0.18	0.08	0.07	0.48	0.47	0.28	0.27	0.01	0.01	0.02	0.01	0.05	0.05	0.01	0.01	1.36	1.37	420	365

For the surrounding normal tissue, the dose at the 1/3 of the volume is, on average, higher for the 6‐MV plan than for the 18‐MV plan, showing the effect of the high entrance dose for the 6‐MV beams. However, the dose at the 2/3 of the normal tissue volume is, on average, lower for the 6‐MV plan than the 18‐MV plan, showing the effect of the high exit dose for the 18‐MV beams. As a result, the 6‐MV and 18‐MV plans yield a nearly identical mean dose to the surrounding normal tissue, as shown in Table 1.

Table 1 also gives the conformal index of all the cases. In general, the conformal index values for the 6‐MV plans are similar to those for the 18‐MV plans and even slightly better in some cases. For most cases, the better conformal index is translated into slightly better rectum sparing.

Table 1 also presents a comparison of the total monitor units between the 6‐MV and 18‐MV plans. On average, the 6‐MV plans deliver 18% more monitor units than do the 18‐MV plans.

## IV. CONCLUSION

In this study, we investigated the feasibility of using 6‐MV intensity‐modulated photons for treating exceptionally large patients with prostate cancer (>30 cm AP or lateral separation). Equivalent dose–volume relationships were found for target volume, rectum, and bladder for either the 6‐MV or 18‐MV intensity‐modulated beams when proper planning techniques were applied. The mean dose to the normal tissue surrounding the target volume was also found to be equivalent for the 6‐MV and 18‐MV beams, which shows that the low entrance dose of the 18‐MV beam is, in effect, balanced by its high exit dose.

Our study shows that the total monitor units were on average 18% higher for the 6‐MV plan as compared with the 18‐MV plan. Because there are no secondary neutrons, and radiation leakage is relatively low for 6‐MV beams, room shielding requirements are significantly less for 6‐MV photons than for 18‐MV photons. The increase in total monitor units can therefore in theory be compensated by increasing the pulse repetition rate of the accelerator by 20% without affecting the workload.

We conclude that a 6‐MV intensity‐modulated beam is a viable and effective option for treating even very large patients with prostate cancer.
